# The physiologic responses to a fluid bolus administration in old and young healthy adults

**DOI:** 10.1186/s13741-022-00266-z

**Published:** 2022-08-16

**Authors:** Cordell Cunningham, Christian Tapking, Michael Salter, Roger Seeton, George C. Kramer, Donald S. Prough, Melinda Sheffield-Moore, Michael P. Kinsky

**Affiliations:** 1 Department of Anesthesiology, The University of Texas Medical Branch, Galveston, TX USA; 2 Department of Surgery, The University of Texas Medical Branch, Galveston, TX USA; 3 Department of Hand, Plastic and Reconstructive Surgery, Microsurgery, Burn Trauma Center, BG Unfallklinik Ludwigshafen, University of Heidelberg, Heidelberg, Germany; 4 Department of Internal Medicine, The University of Texas Medical Branch, Galveston, TX USA

**Keywords:** Fluid response, Cardiovascular, Physiology, Elderly, Older adults

## Abstract

**Background:**

Organ function is known to decline with age. Optimizing cardiac, pulmonary and renal function in older adults has led to significant improvements in perioperative care. However, when substantial blood loss and fluid shifts occur, perioperative outcomes still remains poor, especially in older adults. We suspect that this could be due to age-related changes in endothelial function—an organ controlling the transport of fluid and solutes. The capillary filtration coefficient (CFC) is an important determinant of fluid transport. The CFC can be measured in vivo, which provides a tool to estimate endothelial barrier function. We have previously shown that the CFC increases when giving a fluid bolus resulting in increased vascular and extravascular volume expansion, in young adults. This study aimed to compare the physiologic determinants of fluid distribution in young versus older adults so that clinicians can best optimize perioperative fluid therapy.

**Methods:**

Ten healthy young volunteers (ages 21–35) and nine healthy older volunteers (ages 60–75) received a 10 mL/kg fluid bolus over the course of twenty minutes. Hemodynamics, systolic and diastolic heart function, fluid volumetrics and microcirculatory determinants were measured before, during, and after the fluid bolus.

**Results:**

Diastolic function was reduced in older versus younger adults before and after fluid bolus (*P* < 0.01). Basal CFC and plasma oncotic pressure were lower in the older versus younger adults. Further, CFC did not increase in older adults following the fluid bolus, whereas it did in younger adults (*p* < 0.05). Cumulative urinary output, while lower in older adults, was not significantly different (*p* = 0.059). Mean arterial pressure and systemic vascular resistance were elevated in the older versus younger adults (*p* < 0.05).

**Conclusion:**

Older adults show a less reactive CFC to a fluid bolus, which could reduce blood to tissue transport of fluid. Diastolic dysfunction likely contributes to fluid maldistribution in older adults.

## Key points summary

- Question: Evaluate the physiologic response to fluid bolus in older adults.
- Findings: Capillary filtration coefficient did not increase in older adults.
- Meaning: Older adults appear to retain more fluid compared to younger adults, which is associated with diastolic dysfunction.

## Introduction

Major surgery poses the risk of a patient losing large quantities of blood and fluid. Excessive fluid administration in critically ill patients can lead to cardiac complications (Payen et al. 2008; Boyd et al. 2011; Ranjit et al. 2020). Older adults are particularly vulnerable to fluid shifts (Doherty and Buggy 2012) that occur during major surgery (Pr et al. 1999; Arieff 1999) because they lack physiologic compensatory mechanisms seen in younger adults (Olsen et al. 2000; Docherty 1990; Paneni et al. 2017). This is related to a decline in organ function and age-related maldistribution of electrolytes (Luckey and Parsa 2003; Schlanger et al. 2010). Therefore, large volume fluid replacement therapy must be carefully approached in older patients because fluid mismanagement can lead to organ dysfunction and death (Brandstrup et al. 2003; Nisanevich et al. 2005; Mörgeli et al. 2017; Ko et al. 2019). Despite numerous advances to preoperatively optimize patients to undergo surgery (Mangano et al. 1996; Poldermans et al. 2001), older adults remain at an increased risk for complications of excess fluid (Arieff 1999; Harris et al. 2018; Klein et al. 2007; Miller and Myles 2019).

A reduced conductivity to small solutes and water, measured by a decreased capillary filtration coefficient (CFC), has been described in chronic disease, such as diabetes (Lindenberger and Länne 2007) and is inferred to occur in aging (Gamble et al. 2000). This could be compensatory to prevent fluid loss from the circulation since higher venous pressure and lower venous compliance is prevalent in older adults (Young et al. 1985; Monahan et al. 2001).

We have recently found, in young healthy adults, that the efficiency of a fluid bolus, i.e., how much fluid stays inside the circulation after a fluid bolus, is linked to a rapid but reversible increase in the capillary filtration coefficient (Asmussen et al. 2014). This adaptation may confer protection to excess volume therapy in young healthy patients. This novel finding in healthy young adults has led us to investigate whether CFC fluctuations are an important contributor to fluid transport in older adults.

In this study, we measured the CFC and other hemodynamic variables before, during, and after a fluid bolus in young and older adults. We hypothesize that aging results in a reduced and less responsive CFC during and after a fluid bolus. This increases the potential for older adults to develop volume overload.

## Materials and methods

### Study design

The Institutional Review Board and the General Clinical Research Center Review Board of the University of Texas Medical Branch (UTMB) at Galveston reviewed and approved the protocol and experimental procedures prior to the start of this study (Protocol number 09–215). Inclusion criteria included, men or non-pregnant women in good health, between 21 to 35 years of age and 60 to 75 years of age. Written informed consent was obtained from all subjects and all subjects scored ≥ 26 Mini-Mental State Exam. Subjects were excluded if they had a history of cardiovascular disease determined by history of hypertension, peripheral vascular diseases, heart attack or stroke and previous echocardiographic evidence of diastolic dysfunction (E/e’ > 10) or systolic dysfunction (EF% < 50%). Subjects were also excluded when presenting with cardiac conduction defects, bleeding disorders, anemia, diabetes, neurologic, renal, endocrine or lung diseases (e.g. asthma or chronic obstructive pulmonary disease), known allergic reactions to indocyanine green dye, shellfish or iodine, history of more than 20 pack-years tobacco smoking, alcohol or drug abuse, positive tests for hepatitis or HIV, presence of acute illness or medically unstable chronic illness, or if they were on hormone replacement therapy.

### Study preparation and experimental procedures

This was a prospective, paired, study performed in nineteen healthy volunteers at the General Clinical Research center (GCRC), UTMB. Volunteers were instructed not to eat or drink after midnight, the day before the study. On the day of the study, the volunteers reported to the GCRC. Vital signs (heart rate (HR), non-invasive blood pressure measurement and of peripheral oxygen saturation by plethysmography) and weight were obtained. The subjects were positioned supine in a hospital bed. A three lead ECG, pulse oximeter (Nellcor N600, *Covidien PLC*, Dublin, Ireland) and blood pressure cuff were placed and connected to a clinical monitor (Viridia 24CT, *Hewlett Packard Inc.*, Palo Alto, CA, USA). Two venous catheters (18 G, *B. Braun AG*, Melsungen, Germany) were placed in each arm (either the median cubital vein or a forearm vein) for drug and fluid administration, respectively. An arterial catheter (20 G, Abbocath, *Hospira Inc.*, Lake Forrest, IL, USA) was placed in the radial artery under local anesthesia (1% Lidocaine, 2 mL, *Hospira Inc.*, Lake Forrest, IL, USA) and connected to a pressure monitoring kit (Transpac, *Abbott Laboratories Inc.*, Abbott Park, IL, USA) with a 0.9% NaCl (*Baxter Healthcare Corp.*, Deerfield, IL, USA) pressure bag.

**Fig. 1:**
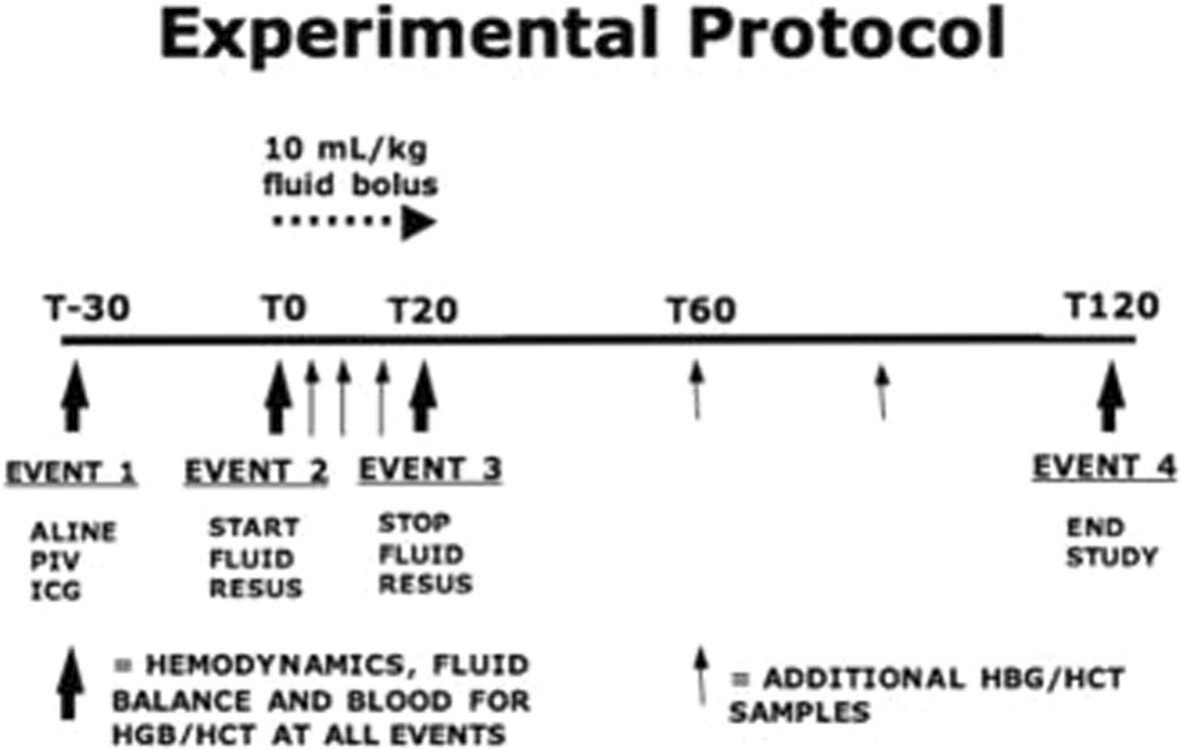
Illustration of experimental protocol

Each subject underwent identical fluid protocols. The specific time points and interventions were (Fig. 1):

- T minus 30 (T-30), after monitors and catheters were placed. Initial plasma volume was determined by indocyanine green.
- T minus 10 (T-10), Measurements were obtained prior to fluid infusion.
- T zero (T0), start of the 10 mL/kg 0.9% saline fluid bolus (*Baxter Healthcare Corp.*, Deerfield, IL, USA)
- T twenty (T20), end of fluid bolus.
- T sixty (T60), 60 min after start of fluid bolus.
- T one hundred twenty (T120), end of study.

Details of the specific measurements and timing are described in detail below.

### Hemodynamic and echocardiography measurements

Measurements for HR and mean arterial blood pressure (MAP) were recorded at T-30, T0, T5, T10, T15, T20, T30, T60, T90 and T120. Echocardiographic (ventricular volume and function) parameters were measured by transthoracic echocardiography using a 3.5 MHz transducer and ultrasound machine (Vivid 7 PRO BT04, *General Electric Medical Systems Inc.*, Milwaukee, WI, USA). Left ventricle (LV) area and length were interrogated in parasternal long axis position. The Modified Simpson’s rule was applied for calculation of end-diastolic (EDV), end-systolic volume (ESV). Stroke volume (SV) was determined from the calculation of EDV – ESV and the ejection fraction (EF%) was determined by SV/EDV. Cardiac output (CO) was calculated from SV • HR. Systemic vascular resistance (SVR) was calculated from CO/MAP. Echocardiographic parameters were measured at T-30 min, T0, T20, T60 and T120. The transthoracic echocardiographies were performed by the senior author, who is a highly experienced anesthesiologist and intensive care physician.

### Volumetric and fluid measurements in mL/kg

The distribution of the 10 mL/kg 0.9% saline fluid bolus was calculated in three compartments (plasma, urine and interstitial volume (extravascular volume)) using mass balance after T0. Cumulative urinary output (UO) was measured via an ultrasound bladder scanner (BVI 3000, *Verathon Inc.*, Bothell, WA, USA). Measurements were obtained at T0, T20, T60, T90 and T120. Initial plasma volume (PV) was determined with the spectrophotometric detection of indocyanine green (ICG, *Akron Inc.*, Lake Forrest, IL, USA) using optical densitometry bound to plasma proteins (Henschen et al. 1993). Specifically, 5 mg ICG was injected intravenously at T-15. Blood samples were taken every minute for six minutes. The amount of ICG bound to plasma proteins (PV_ICG_) was measured at 840 nm using spectrophotometry (DU 800, Beckman Coulter Inc., Brea, CA, USA). The change in PV (ΔPV) is directly proportional to the initial plasma volume + changes in hematocrit (*ΔPV* ~ *PV*_*i*_ + *ΔHematocrit *_*after/initial*_*) (*Henschen et al. 1993). Arterial blood for hemoglobin and hematocrit were taken prior to, during and after the fluid bolus to calculate vascular volume expansion. Specifically, samples were obtained at start of fluid bolus and every two minutes during the fluid bolus, every five minutes until T60 and then every 30 min until T120. Plasma Volume (PV) along with hemoglobin/hematocrit at specific time points was used to calculate PV expansion that occurred after the fluid bolus. Change in extravascular volume (EVV) over time (ΔEVV) was calculated from UO, plasma volume and total fluid administered: *ΔEVV* = *infused volume—(ΔPV* + *UO* + *fluid infused)* (Stephens et al. 2011; Kinsky et al. 2008).

### Microcirculatory measurements

**Fig. 2:**
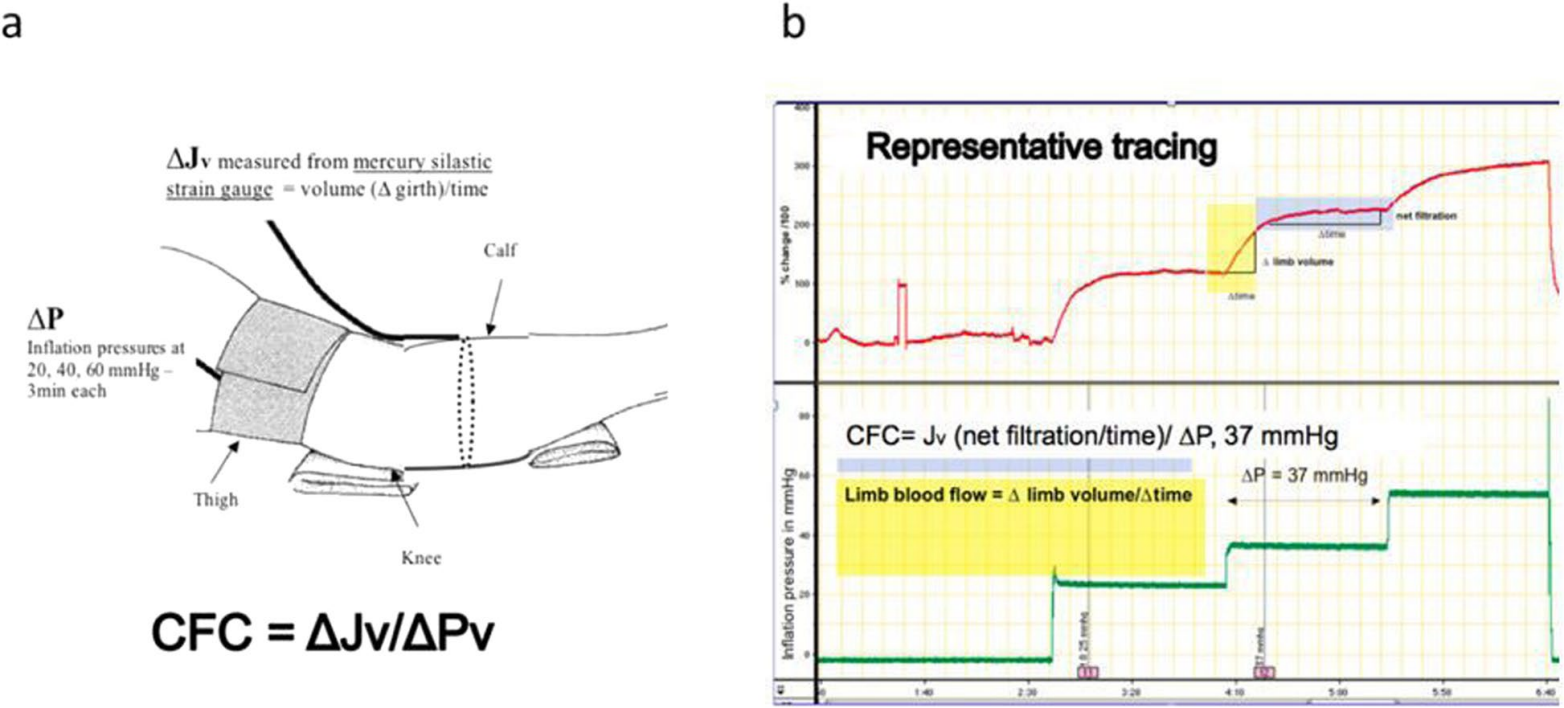
Capillary filtration coefficient measurement: Schematic of patient setting with mercury silastic stain gauge (**a**) and sample of measured results and calculation (**b**)

The capillary filtration coefficient (CFC) was determined in vivo using venous congestion plethysmography (VCP). In brief, step-wise increases in venous pressure were performed by applying a pressurized cuff on the thigh and a mercury-silastic strain gauge transducer on the calf (Hokanson EC6 strain gauge plethysmograph, *D.E. Hokanson Inc*., Bellevue, WA, USA) (Fig. 2a) (Gamble et al. 1993; Gamble et al. 1992; Christ et al. 1993; Christ et al. 1995). The change in limb volume/girth, measured by the strain gauge transducer, represents the net fluid filtration or Jv (in which each % = 1 mL/100 mL tissue) if the exceeded filtration pressure of Pv (via the pressurized cuff in mmHg) is held for several minutes. Thus, CFC was determined from the % girth over time (net fluid filtration (∆J_v_)) divided by the changes in venous pressure (∆P_v_) as: CFC = ∆J_v_/∆P_v_. Three separate inflation pressures (~ 30, 45 and 60 mmHg), each sustained for three minutes, were performed before fluid bolus, during and immediately at end of fluid bolus, one hour after fluid bolus and study end (T-30, T-10, T0, T20, T60 and T120). The collection of these measurements provided insight into derangements in the Starling Equation, as described below.

The amount of pressure applied to the cuff, limb girth % and time (min) were digitally sampled and stored using Powerlab (ADInstruments Inc., Colorado Springs, CO, USA). The CFC was determined from an offline analysis using the shallow slope from the change of % limb girth and time (Fig. 2b). VCP measurements at each time point took approximately nine to twelve minutes to complete.

Blood samples, taken at the same time points as CFC measurements, for total protein and albumin were analyzed using a protein analyzer (Vitros Fusion 5.1, Ortho Clinical Diagnostics Inc., Raritan, NJ, USA). The plasma colloid osmotic pressure (COP_pl_) was estimated using a derived formula from these constituents as previously described in detail (Kinsky et al. 1998a; Navar and Navar 1977). A full list of measured variables can be found in Table 1.

**Table 1:** List of measured variables in this study

Variable	Unit	Description
Mean arterial pressure (MAP)	mmHg	Average blood pressure in an individual during a single cardiac cycle
Heart rate (HR)	bpm	Number of contractions of the heart per minute
Cardiac Output 9CO)	L/min	Product of heart rate and stroke volume
Systemic Vascular Resistance (SVR)	(dyne*sec/cm.^5^)	The resistance to blood flow offered by all of the systemic vasculature, excluding the pulmonary vasculature
Enddiastolic Volume (EDV)	mL	Volume of blood in the right and/or left ventricle at end load or filling in (diastole) or the amount of blood in the ventricles just before systole
Endsystolic Volume (ESV)	mL	Volume of blood in a ventricle at the end of contraction
Diastolic Function	E/e’	Filling of the heart
Systolic Function	Ejection Fraction (%)	Volumetric fraction of fluid (usually blood) ejected from a chamber with each contraction
Urinary Output (UO)	mL/kg	Volume of urine per kilogram body weight and time
Extravascular Volume (EVV)	mL/kg	Fluid volume expansion that occurs outside of the vascular system
Plasma Volume	mL/kg	Total volume of blood
Capillary Filtration Coefficient (CFC)	mL/min/mmHg	Reflects both microvascular hydraulic conductivity and the number of perfused capillaries at a given moment
Colloid Osmotic Pressure in Plasma	mmHg	Form of osmotic pressure induced by proteins, notably albumin, in a blood vessel's plasma (blood/liquid) that causes a pull on fluid back into the capillary

### Statistical analysis

Power calculation: Our statistical hypothesis was that the capillary filtration coefficient (CFC) would be reduced and less responsive in older subjects. Therefore, sample size determination was based on the primary outcome, basal CFC. Data obtained in previous young healthy volunteers showed that CFC was 2.6 ± 1.0 (mean ± SD). We surmised that older adults would result in a 50% lower CFC, based on lower transcapillary fluid reabsorption rates reported in older adults. A sample size of 10 per group would have 80% power to detect an effect size of 1.000 using a two-group t-test with a 0.050 two-sided significance level. Thirteen subjects in each group were recruited to accommodate for attrition.

Students t-test and two-way analysis of variance were used for data analysis. Bonferroni post hoc comparison was used when applicable. Statistical analyses were performed using Prism 4 for Mac (GraphPad, Software Inc, La Jolla, USA). Data are expressed as mean ± S.E.M. Statistical significance was accepted when p ≤ 0.05.

## Results

### Baseline characteristics

**Fig. 3:**
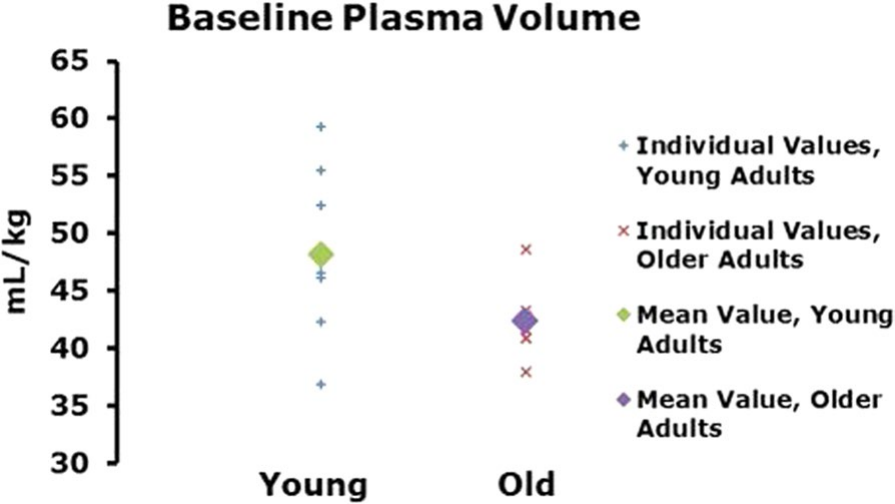
Baseline plasma volume in young versus old subjects

Baseline plasma volume was not significantly lower in the older population (Δ 5.85 mL/kg, Fig. 3).

### Hemodynamics (Table 2) and cardiac function

**Table 2:** Hemodynamics before, during and after 0.9% NaCl 10 mL/kg fluid bolus

	**Time**	**Baseline**	**Pre-Bolus**	**Post-Bolus**	**Study End**
		**M ± SEM**	**M ± SEM**	**M ± SEM**	**M ± SEM**
**Mean Arterial Pressure (mmHg)**	**Younger**	82 ± 3	83 ± 2	83 ± 3	80 ± 2
	**Older**	90 ± 2	90 ± 2	87 ± 3	92 ± 4
**Heart Rate (bpm)**	**Younger**	66 ± 3	63 ± 2	65 ± 1	64 ± 3
	**Older**	66 ± 4	61 ± 4	67 ± 3	62 ± 3
**Cardiac Output (L/min)**	**Younger**	5.3 ± 0.2	5.2 ± 0.1	6.3 ± 0.3	5.4 ± 0.2
	**Older**	5.5 ± 0.3	5.1 ± 0.3	6.5 ± 0.3	5.3 ± 0.3
Systemic Vascular Resistance (dyne*sec/cm.^**5**^**)**	**Younger**	1246 ± 57	1265 ± 34	1062 ± 48	1195 ± 28
	**Older**	1348 ± 73	1445 ± 77	1079 ± 51	1404 ± 83

*M* Mean, *SEM* Standard error of the mean, bpm – beats per minute, *sec* second, *min* minute, *L* liter, *cm* centimeter, *dyn* dyne

Baseline – 30 min before fluid bolus, pre-bolus – immediately before fluid bolus, post-bolus – immediately after fluid bolus, study end – 120 min after start of fluid bolus

HR and CO were similar during all time points throughout the study. CO reached its highest peak at the end of the fluid bolus. MAP was elevated in the older population before, during, and after the fluid bolus (*p* < 0.001). SVR was elevated in the older population (*p* < 0.05), although both populations had a similar SVR directly after the fluid bolus. Also, both groups showed a similar drop immediately at the end of the fluid bolus administration.

**Fig. 4:**
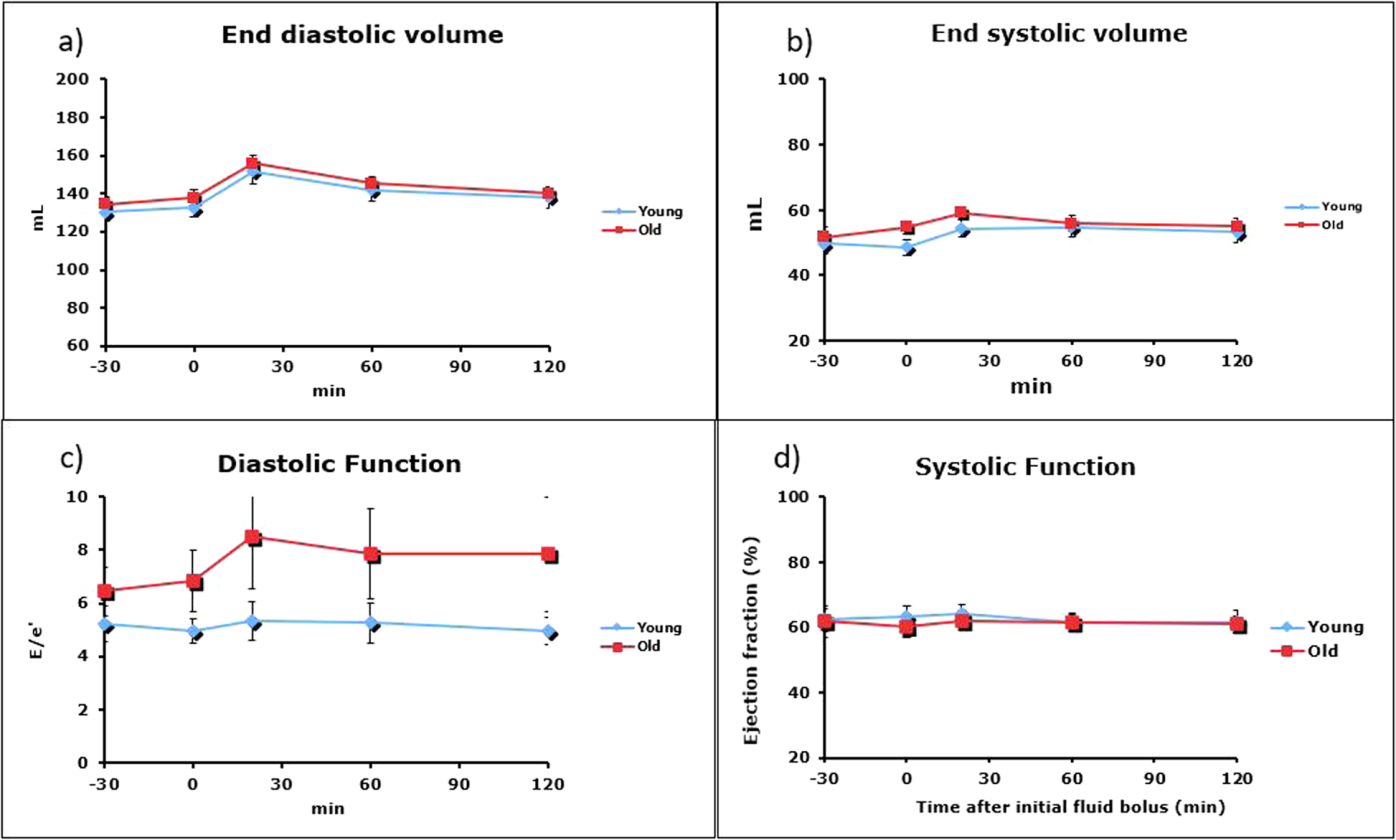
End Diastolic Volume (**a**), End Systolic Volume (**b**), Diastolic Volume (**c**) and Systolic Function (**d**) before and after fluid bolus

End-diastolic volume (EDV) was not significantly different between the groups (Fig. 4a). The fluid bolus was associated with a small increase in End-systolic volume (ESV). ESV was significantly elevated throughout the study in the older population (*p* < 0.05, Fig. 4b). Diastolic function (E/e’) was reduced in the older adults throughout the entirety of the study (*p* < 0.001), and continued to rise during and after the fluid bolus (Fig. 4c). This shows significant dysfunction compared to the younger population. Systolic function (Ejection Fraction %) was similar in both groups and slightly increased following the fluid bolus (Fig. 4d).

**Fig. 5:**
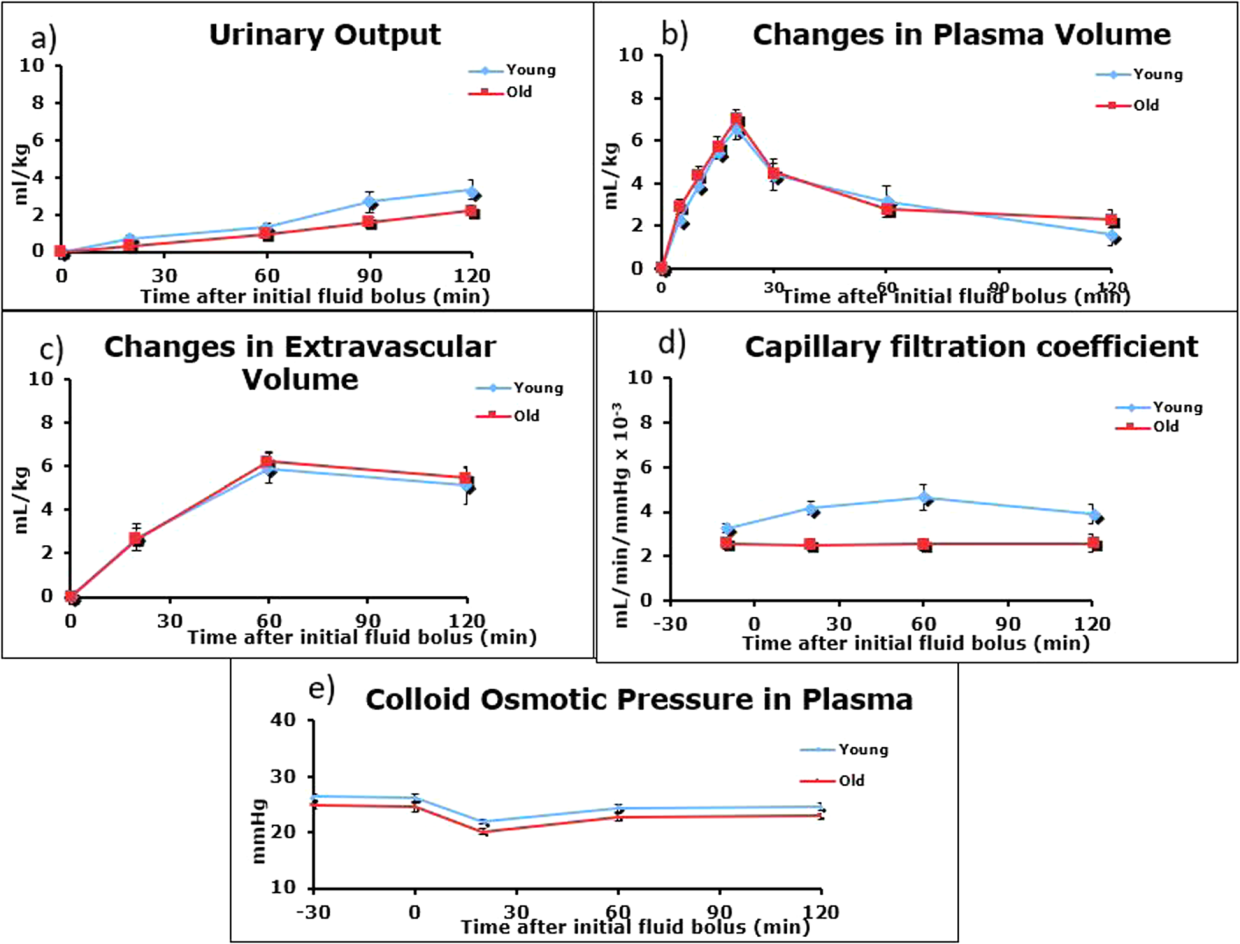
Urinary Output (**a**), Volume expansion (**b** and **c**) after fluid bolus*,* Capillary Filtration Coefficient (**d**) and Colloid Osmotic Pressure in Plasma (**e**)

Cumulative Urinary Output (UO) was lower in the older population compared to the younger population, though this result was not statistically significant (*p* = 0.059, Fig. 5a). The fluid bolus resulted in a transient peak in both groups immediately at the end of the bolus. Plasma volume was similar in both groups. This expansion did not completely resolve by the end of the study. The fluid bolus resulted in extra-vascular expansion (EVV), which was similar for both groups. Vascular volume efficiency (VVE – calculated as ΔPV divided by fluid_in over time_) was similar in both populations (Fig. 5b and c).

### Microcirculatory determinants

The CFC was not related to the fluid bolus in the older population (Fig. 5d). At every time point, the fluctuation from baseline was small and insignificant. In the younger population, the CFC was higher at all points throughout the study (*p* < 0.05). After rising to a level that was significantly different (T60 from T20), CFC levels in the younger population fell slightly towards baseline values during/after the fluid bolus (T120) All timepoints were significantly different from baseline in the younger population. The fluid bolus resulted in no significant change from baseline in the older population. COP_pl_ significantly fell in both groups after the fluid bolus. The nadir occurred at 20 min. There was partial restoration in COP_pl_ to basal levels by study end (Fig. 5e).

## Discussion

Since organ function tends to decline with advanced age, perioperative fluid therapy can be challenging in older adult patients, which increases the risk for complications for fluid mismanagement (under and over resuscitation). This study evaluated physiologic differences in older adults that govern the distribution of a fluid bolus.

Cardiovascular and blood volume differences, based on age during non-dynamic conditions, have been reported. Specifically, Best et al., showed that older subjects have a lower blood volume and stroke volume, and higher systemic vascular resistance (SVR) (Best et al. 2014). Our data supports these findings. We observed basal plasma volume was reduced by 10% in older adults compared to younger adults. Our data also showed that SVR at baseline and 120 min after a fluid bolus was increased in older adults. Our study evaluated differences in diastolic function during and after fluid bolus, which showed that the fluid bolus in older adults resulted in impaired relaxation or diastolic dysfunction (E/e′ > 8), suggesting that older adults have a reduced ability to adapt to rapid fluid expansion.

A key finding in this study was the reactivity in the CFC between older and younger adults before and after the fluid bolus. Interestingly, while older adults had a lower CFC, vascular and extra-vascular volume expansion after the fluid bolus was not increased compared to younger adults. This is somewhat paradoxical in that reduced CFC should expand plasma volume due to reduced blood-to-tissue transport; on the other hand, reduced diastolic function increases the filling pressure of the heart. Increased filling pressure could increase the capillary pressure (P_c_) and increase the net fluid movement (Jv). This relationship can be understood through the Starling equation, which describes the forces that determine fluid movement across capillaries:

$$\mathrm{Jv}=\mathrm{CFC}\left[\left({\mathrm P}_{\mathrm c}-{\mathrm P}_{\mathrm{if}}\right)-\mathrm\sigma{\textstyle\prod_{\mathrm p}}-{\textstyle\prod_{\mathrm{if}}}\right]$$

Here, Jv represents the fluid filtration, CFC is capillary filtration coefficient, P_c_ is the capillary pressure, P_if_ is interstitial hydrostatic pressure, σ is the reflection coefficient for protein, Π_p_ is plasma colloid osmotic pressure and Π_if_ interstitial colloid osmotic pressure.

This mechanism of an unresponsive CFC in the older adults, coupled with an impaired diastolic function, which increases the filling pressure in the central veins, might explain why the ΔPV was nearly identical between the two groups and why the baseline PV was lower in the older adults. Additionally, lower plasma colloid osmotic pressure in older adults likely contributed. There are several other microcirculatory determinants that were not account for but important. For example, interstitial compliance (measured by interstitial pressure and volume) is reduced in aging and could contribute to volume trapping in older adults (Sarbacher and Halper 2019).

Finally, other fluid/volumetric (compensatory) responses likely contribute to the physiologic responses after a fluid challenge. Specifically, changes in splanchnic circulation and response to hypo- and hypervolemia must be recognized. The splanchnic circulation is a low resistant high capacity system that receives approximately 25% of the cardiac output and therefore serves as an important fluid and blood reservoir (Greenway and Lister 1974). Little is known about the age-related changes in splanchnic circulation. However, it seems plausible that changes in the reservoir function of the splanchnic system can be linked to the response to fluid bolus or fluid overload. The splanchnic system may be more capable of adjusting to fluid overload in younger subjects. Hormonal changes due to aging require further study. It is recognized that total body water, fluid intake and thirst is reduced in older adults (McKinley et al. 2006). Thus, the impact of fluid resuscitation and its mismanagement (hypo – and hyper volumia) on the macro and microcirculatory would likely be more exaggerated in this age group.

### Limitations

We acknowledge that this study has several limitations. Our sample size was small and slightly imbalanced (*n* = 9 and *n* = 10 for older versus younger adults, respectively). Also, the volume challenge or fluid bolus (10 mL/kg) was modest in this study, mostly due to safety reasons. Previously, our group showed more pronounced changes in CFC, in younger adults, with a 25 mL/kg fluid bolus (Asmussen et al. 2014). However, we still saw a dynamic CFC in the younger adults during this study. It is unclear whether or not a larger fluid bolus would have any association with CFC in older adults. On the other hand, a larger size bolus would likely produce greater changes in vascular and extravascular expansion. This could explain lack of statistical differences between groups.

We focused on the CFC and COP_pl_ as determinants of transvascular fluid flux. These methods were chosen due to their reproducibility, ease of measurement and validity in clinical subjects (Gamble et al. 1993; Gamble et al. 1992; Christ et al. 1993). Evaluation of CFC using venous congestion plethysmography (VCP) requires the subject to remain still for several minutes at each time point, which was possible in our healthy volunteers, but could be problematic in patients that are shivering or are non-cooperative. Therefore, it would be difficult to conduct these assessments on patients being treated for trauma/hemorrhage. This study also excluded older adults with disease and illness, although this subset of patients is at a much higher risk for complications associated with excess fluid. VCP is an indirect assessment of the CFC. Direct CFC measurements require small vessel cannulation, which would be invasive and impractical in awake volunteers. Additionally, we used albumin and total protein to estimate the COP_pl_, as previously reported (Navar and Navar 1977; Kinsky et al. 1998b). The COP_pl_ is directly related to circulating macromolecules. Other endogenous proteins are essentially non-contributory to oncotic pressure. Since we did not administer exogenous fluid solutions with larger molecules, such as starch or dextran, we assumed that the serum protein and albumin concentration reflected COP_pl_ at each time point. Other microciculatory determinants such as capillary pressure, interstitial hydrostatic and oncotic pressure and reflection coefficient were not measured. Direct P_c_ requires invasive cannulation and isolation. Indirect measurements such as venous pressure are now being pursued but were not measured in these volunteer studies. Other Starling variables such as interstitial hydrostatic and oncotic pressure and protein reflection coefficient requires non-movement (ideally paralysis) and invasive techniques that were not practical for healthy volunteer studies. Finally, we did not measure total body water or extracellular water and other hormones, which could have impacted retention and fluid distribution.

## Conclusion

This study showed that the CFC in older versus younger adults was less reactive to an intravenous fluid bolus. Older adults tend to retain more fluid than younger adults, but not in the plasma. Although a lower CFC by itself would decrease transcapillary fluid etravasation during the fluid bolus, impaired diastolic function likely offsets this interaction. This mechanism may explain lower basal plasma volume yet similar plasma volume expansion in the two age groups. This may also contribute to fluid trapping, such as the lower urinary output seen in the older adults. Additionally, the association a larger fluid bolus and other factors need further investigation.

## Data Availability

The dataset used in this study is available from the corresponding author on reasonable request.

## Abbreviations

CFC: Capillary filtration coefficient
CO: Cardiac output
COP: Colloid osmotic pressure
EDV: End-diastolic volume
EF: Ejection fraction
ESV: End-systolic volume
EVV: Extravascular volume
HR: Heart rate
LV: Left ventricle
MAP: Mean arterial pressure
PV: Plasma volume
SV: Stroke volume
UO: Urinary output
VCP: Venous congestion plethysmography
